# Supplementary material for: Vascular Endothelial Cell-Derived Exosomal Sphingosylphosphorylcholine Attenuates Myocardial Ischemia–Reperfusion Injury through NR4A2-Mediated Mitophagy
Source: Int J Mol Sci. 2024 Mar 14;25(6):3305. doi: 10.3390/ijms25063305 (PMC10970187; doi:10.3390/ijms25063305)
Supplement: Supplementary file 1 [file ijms-25-03305-s001.zip › Supplementary materials (Figure S1).pdf]

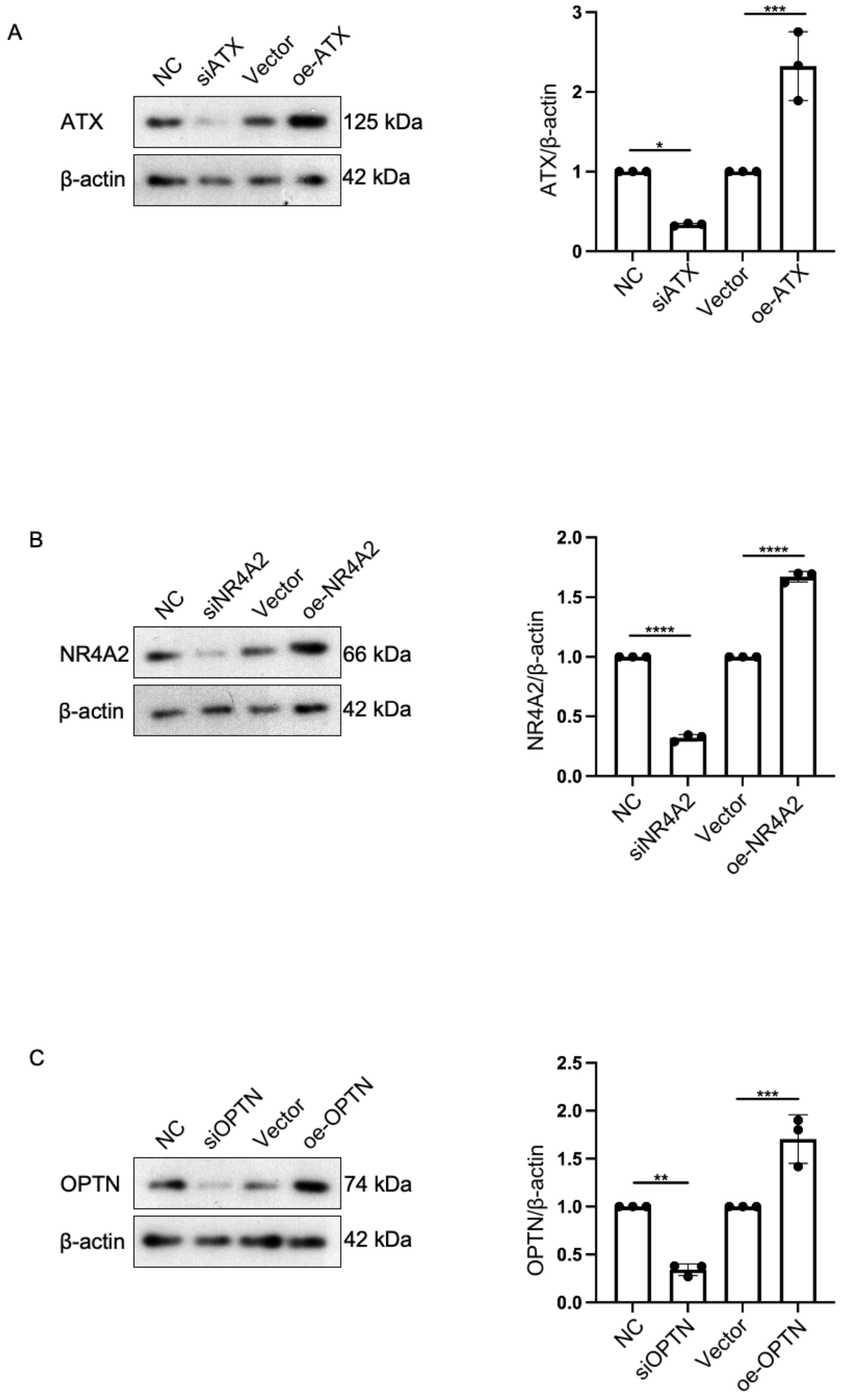

Figure S1. Validation of transfection effect of siRNA and overexpression plasmids for ATX, NR4A2 and OPTN. (**A**) Validation of transfection effect of siRNA and overexpression plasmid of ATX. (**B**) Validation of transfection effect of siRNA and overexpression plasmid of NR4A2. (**C**) Validation of transfection effect of siRNA and overexpression plasmid of OPTN. In all statistical graphs, $n = 3$; * $p < 0.05$, ** $p < 0.01$, *** $p < 0.001$, and **** $p < 0.0001$.
